# The Use of an Edible Mushroom-Derived Renewable Carbon Material as a Highly Stable Electrocatalyst towards Four-Electron Oxygen Reduction

**DOI:** 10.3390/ma9010001

**Published:** 2015-12-23

**Authors:** Chaozhong Guo, Lingtao Sun, Wenli Liao, Zhongbin Li

**Affiliations:** 1 Research Institute for New Materials Technology, Chongqing University of Arts and Sciences, Yongchuan 402160, Chongqing, China; ltsun@cqwu.edu.cn; 2 College of Materials and Chemical Engineering, Chongqing University of Arts and Sciences, Yongchuan 402160, Chongqing, China; lzb@cqwu.net

**Keywords:** enoki mushroom, electrocatalyst, oxygen reduction, carbon material

## Abstract

The development of highly stable and efficient electrocatalysts for sluggish oxygen reduction reaction (ORR) is exceedingly significant for the commercialization of fuel cells but remains a challenge. We here synthesize a new nitrogen-doped biocarbon composite material (N-BC@CNP-900) as a nitrogen-containing carbon-based electrocatalyst for the ORR via facile all-solid-state multi-step pyrolysis of bioprotein-enriched enoki mushroom as a starting material, and inexpensive carbon nanoparticles as the inserting matrix and conducting agent at controlled temperatures. Results show that the N-BC@CNP-900 catalyst exhibits the best ORR electrocatalytic activity with an onset potential of 0.94 V (*versus* reversible hydrogen electrode, RHE) and high stability. Meanwhile, this catalyst significantly exhibits good selectivity of the four-electron reaction pathway in an alkaline electrolyte. It is notable that pyridinic- and graphtic-nitrogen groups that play a key role in the enhancement of the ORR activity may be the catalytically active structures for the ORR. We further propose that the pyridinic-nitrogen species can mainly stabilize the ORR activity and the graphitic-nitrogen species can largely enhance the ORR activity. Besides, the addition of carbon support also plays an important role in the pyrolysis process, promoting the ORR electrocatalytic activity.

## 1. Introduction

High-performance fuel cells and metal-air batteries are a class of promising green power sources and their rapid development become a key solution to solving the energy shortage and environmental problems. However, the oxygen reduction reaction (ORR) at the cathode produces many negative effects on the battery systems owing to its sluggish dynamic behavior and diversity of reaction pathways [1,2]. Carbon-supported Pt nanoparticles are widely used as the most efficient catalysts due to their high ORR activity, but high cost and resource scarcity of metal Pt as well as poor durability hinder their sustainable and commercial applications [3]. Therefore, facile synthesis of non-Pt ORR catalysts such as non-precious-metal catalysts (NPMCs) and metal-free catalysts (MFCs) to replace expensive Pt-based catalysts recently became a topic of interest.

In the past decades, various non-Pt catalysts, including NPMCs [4,5,6], transition-metal oxides (TMOs) [7], and heteroatoms-doped carbon materials (HDCMs) [8] were effectively developed [5]. More notably, the HDCMs attracted more attention and were considered as a very promising ORR electrocatalyst due to their unique structures and excellent electrocatalytic performances [9]. The HDCMs such as nitrogen-doped graphene [10], nitrogen-doped carbon nanotubes [11] and nitrogen-doped carbon microspheres [12] have exhibited reasonable ORR activity and durability, and the catalytic mechanism of HDCMs and their catalytically active sites are not clearly defined. It is certain that heteroatoms are introduced into the carbon structure by *in-situ* or post-doping methods, which can largely improve the ORR activity. It may be mainly attributed to the difference in bond length, valence electron and atomic size between carbon and heteroatom [13,14]. Recently, the synthesis of a nitrogen-doped biocarbon (N-BC) catalyst was originally performed by the rapid pyrolysis of hemoglobin at controlled temperatures [15], which demonstrated the possibility of bioprotein for use in the design of HDCMs at certain conditions. Subsequently, lots of research has utilized biomass-containing bioprotein as the nitrogen precursor or starting material of the doped carbon catalysts with high electrocatalytic activity and outstanding durability [16,17,18,19,20,21,22]. We also formed several nitrogen-containing NPMCs based on animal protein-rich blood waste by using the “all-solid-state multi-step pyrolysis method”, and further confirmed the active sites for the ORR with the help of modern physical techniques only [23,24].

Enoki mushroom (EM) are widely distributed in China, Japan, Russia, Australia, North America and other regions, and they are a famous food and edible fungus all over the world because of their high nutritional value and medicinal value. Yuan *et al.* [25] have specially detected the contents of various nutrients in dried EM biomass, and confirmed that the mass content of biological protein exceeds 31.0 wt %, which is higher than that of pig blood waste [24]. Herein, we propose an easy strategy for the synthesis of N-doped carbon catalysts for ORR (N-BC@CNP-900) via using EM as a starting precursor and inexpensive carbon nanoparticles (CNP) as the conducting agent and inserting matrix during pyrolysis under the nitrogen atmosphere. The ORR activity and long-term stability of this catalyst under alkaline and acidic conditions were adequately evaluated, and its internal structures were systematically characterized. To the best of our knowledge, sustainable development of highly stable doped carbon catalysts from economical mushroom biomass has scarcely been reported.

## 2. Experimental Section

### 2.1. Material Synthesis

First, raw EM biomass supplied by the Food Testing Center of Chongqing Bureau of Quality and Technology Supervision was absolutely dried at 120 °C in a vacuum drying oven, and then ground in an agate mortar for about 2 h. The dried EM powder was further carbonized at 300 °C for 5 h in order to promote the decomposition of biological protein as far as possible. The selection of carbonization temperature mainly referred to the thermogravimetric analysis curve of EM dried powder (Figure S1). The decomposition of bioprotein inside EM is easy to obtain at 285 °C. We can find that the thermal decomposition of bioprotein inside EM occurred at ~300 °C, and the obtained carbonaceous material was marked for EM300. Subsequently, 0.4 g of EM300 and 0.2 g of carbon nanoparticles (Vulcan XC-72R, Shanghai Hesen Electrical Co., Ltd., Shanghai, China, 30 nm diameter) were adequately mixed by ball-milling for 5 h at 400 rpm to produce the N-BC@CNP precursor. The N-BC@CNP was heat-treated in a tubular furnace at 700, 800, 900 and 1000 °C for 2 h, respectively. The samples were labeled as N-BC@CNP-700, N-BC@CNP-800, N-BC@CNP-900, and N-BC@CNP-1000, respectively. As a control, direct pyrolysis of EM dried powder at 900 °C was carried out to obtain the N-BC-900 sample. Another sample (N-BC-300900) was also prepared by two-step carbonization of EM dried powder at 300 °C and 900 °C for 2 h, respectively [26]. All the heat-treatment processes were carried out in nitrogen atmosphere with a heating rate of 10 °C·min^−1^.

### 2.2. Physical Characterization

Powder X-ray diffraction (XRD) patterns of the samples were performed on a Shimadzu XRD-6000 X-ray diffractometer (Shimadzu Ltd., Kyoto, Japan) using Cu Kα_1_ radiation (λ = 1.5406 Å) at 4°·min^−1^. X-ray photoelectron spectroscopy (XPS) analysis was studied using a VG Scientific ESCALAB 220 iXL spectrometer (VG Scientific, St Leonards-on-Sea, UK) with an Al Kα (*hv* = 1486.69 eV) X-ray source. The surface and morphology of the samples were visualized using electron microscopy facilities (S-4700 high-resolution microscope operated at an acceleration voltage of 10 kV (Hitachi Ltd., Hitachi, Japan) and Libra200FE high-resolution microscope operated at 200 kV (Carl Zeiss GmbH, Jena, Germany)). Thermogravimetric and differential thermogravimetric (TG-DTG) analysis of EM dried powder was carried out on a Shimadzu differential thermal analyzer (DTG-60H, Shimadzu Ltd., Kyoto, Japan) under N_2_ flowing. 

### 2.3. Electrochemical Measurements

Electrochemical experiments were conducted at room temperature on a Zahner Zennium-E (Kronach, Germany) electrochemical workstation. A ring-shaped Pt wire and a saturated calomel electrode (SCE) were used as the counter and reference electrodes, respectively. A rotation disk electrode (RDE) with a glass carbon (GC) electrode (4 mm diameter, American Model 636 Princeton Applied Research, 0.1256 cm^2^ geometric area) was used as the working electrode. The electrolyte was 0.1 mol·L^−1^ KOH solution or 0.5 mol·L^−1^ H_2_SO_4_ solution. The modified-GC working electrode was fabricated by coating it with catalyst ink. Typically, 5 μL catalyst ink, well-dispersed by 1.0 wt % nafion/isopropanol solution, was dropped onto the GC-RDE surface and then dried in air. About 50 μg of the catalysts, except 40% Pt/C (10 μg), was loaded on the GC disk electrode. All of the electrode potentials in this study are quoted *versus* a reversible hydrogen electrode (RHE). The modified-GC electrode was activated by voltammetric scanning over the potential range of 0.1 to 1.2 V (*vs.* RHE) for 20 cycles at a scan rate of 50 mV·s^−1^ in N_2_-saturated electrolytes. All RDE experiments for ORR were performed by cyclic voltammograms (CVs) or linear sweep voltammograms (LSVs) over the same potential range at a scan rate of 5 mV·s^−1^ in O_2_-saturated electrolytes under the rotation rate from 400 to 3600 rpm. The durability of the prepared catalyst was evaluated using an accelerated aging test (AAT). The AAT uses 2000 continuous potential cycles performed by cyclic scanning potential between 0.1 and 1.2 V in O_2_-saturated alkaline and acidic solutions.

## 3. Results and Discussion

### 3.1. Structural and Surface Characterization

**Figure 1:**
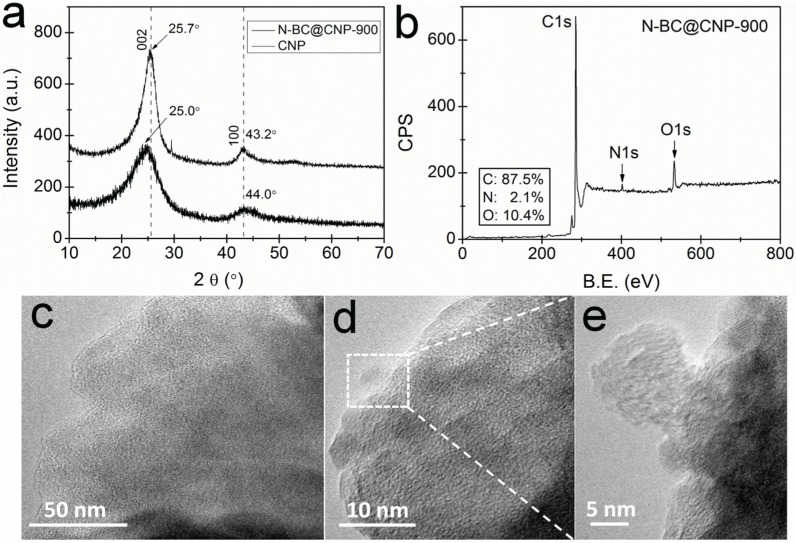
(**a**) The X-ray diffraction (XRD) patterns of carbon nanoparticles (CNP) and N-BC@CNP-900; (**b**) X-ray photoelectron (XP) full-scan spectrum of N-BC@CNP-900; B.E.: Bonding energy; (**c**–**e**) High-resolution transmission electron microscopy (HR-TEM) images of N-BC@CNP-900.

The XRD analysis was applied to study the structural change of the carbon material on the basis of the doping of heteroatom-nitrogen. Figure 1a displays wide-angle XRD patterns of CNP and N-BC@CNP-900 with two broad peaks centered at ~25° and ~44.0°, corresponding to the 002 and 100 lattice planes of a typical amorphous carbon [23]. The interlayer distance (*d*-spacing) between the stacks of carbon nanosheets is higher in turbostratic carbon than in CNP along with a higher degree of disorder [27]. The full width-half maximum (FWHM) of the 002 peak for CNP is also smaller than that for N-BC@CNP-900, which is attributed to an increase in the grain size. In addition, the increase in the FWHM of the 002 carbon peak for CNP is indicative of the nitrogen atom to be incorporated into the carbon structure [24]. The 002 carbon peak of N-BC@CNP-900 was also shifted to lower 2θ value compared to the CNP. This is due to the slight distortion in crystalline regularity along the *a* or *b* direction by the introduction of N atoms in the sp^2^ carbon lattice [24]. The above results indicate that the CNP was modified with N-rich decomposed fragments of EM300 during pyrolysis at high temperatures.

The X-ray photoelectron (XP) full-scan spectrum of N-BC@CNP-900 was carried out in order to investigate its surface composition and chemical bonding, as shown in Figure 1b. It is observed that C1s, N1s and O1s can be distinctly recognized from the XPS survey scan, and their contents are 87.5, 2.1 and 10.4 at %, respectively. The presence of the N1s peak indicates that N atoms are successfully incorporated into the graphite structure of N-BC@CNP-900. In addition, the XPS peaks of trace transition metals are not found in Figure 1b, suggesting that the content of transition metals should be below the detection limit of the XPS testing technique, which is supported by XRD analysis in Figure 1a. We further tested the transition metal contents of the N-BC@CNP-900 by the inductive coupled plasma (ICP) emission spectrometer. It can be found that the contents of Fe and Co are 0.26 and 0.23 wt %, respectively, which may mainly come from impurities of CNP and EM300. The structure and morphology of N-BC@CNP-900 were characterized by scanning electron microscopy (SEM, Hitachi Ltd.) and high-resolution transmission electron microscopy (HR-TEM, Carl Zeiss GmbH), as displayed in Figure S2 and Figure 1c–e, separately. Some massive particles (N-doped BC) were formed during pyrolysis due to the existence of plant cellulose in EM300, and were also covered and enwrapped by N-doped CNP (see Figure S2). The HR-TEM image of N-BC@CNP-900 (Figure 1c) demonstrates the amorphous carbon structure without conspicuous pore structures, but many disordered defects can be observed in the edge regions of graphite layers thanks to the structure distortions caused by the doping of N atoms into graphite lattices (Figure 1d,e). The defected structures and their relative N-bonding configurations exposed on the surface of the carbon material will be closely related to the ORR activity [24].

**Figure 2:**
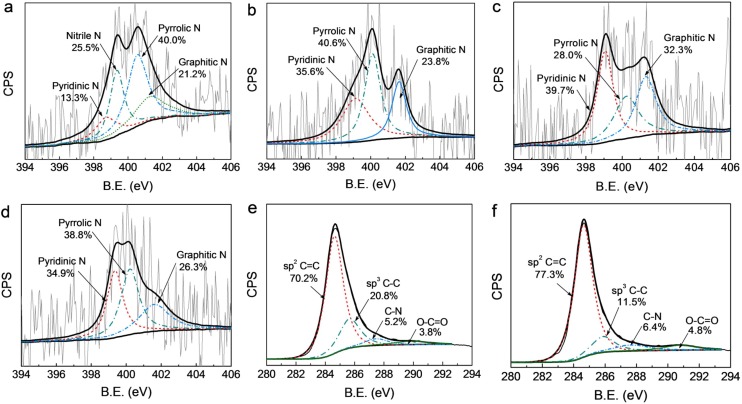
High-resolution N1s XP narrow-scan spectra of EM300 (**a**); N-BC@CNP-700 (**b**); N-BC@CNP-900 (**c**); and N-BC@CNP-1000 (**d**); High-resolution C1s XP narrow-scan spectra of EM300 (**e**) and N-BC@CNP-900 (**f**).

The chemical status of nitrogen atoms in the doped carbon materials including EM300, N-BC@CNP-700, N-BC@CNP-900 and N-BC@CNP-1000 was probed by high-resolution N1s narrow-scan XP spectra (Figure 2a–d). The overall nitrogen content, which was determined via elemental analysis, was 5.3 at % in EM300 but was only 2.4, 2.1 and 1.9 at % in N-BC@CNP-700, N-BC@CNP-900, and N-BC@CNP-1000, respectively. This result suggests that further pyrolysis of EM300 at high temperatures can induce an obvious loss of total N content but enrich more carbon sources due to the evolution of nitrogen-based biomolecules inside EM300. However, the high-temperature process gives rise to the wastage of the CNP support, because it can be partially oxidized by the oxygen from the decomposed products of EM300, further confirmed by our previous results [24]. It is concluded that the total N content of nitrogen-doped carbon materials decreases with the increase of the pyrolysis temperature, whereas the amount of N-doping in as-prepared samples may be not reduced. Take EM300 and N-BC@CNP-900 as typical examples to display the results in Figure 2e,f. The C1s peak of N-BC@CNP-900 at 284.6, 285.7, 287.6 and 290.8 eV (Figure 2f) can be assigned to graphitic sp^2^ C=C, amorphous sp^3^ C–C, sp^2^ carbon atoms bonded to nitrogen (sp^2^ C=N) and sp^2^ carbon atoms bonded to oxygen (O–C=O), with relative proportions of 77.3, 11.5, 6.4 and 4.8 at %, respectively. Interestingly, we can find that the relative percentage of sp^2^ C=N in N-BC@CNP-900 is obviously higher than that of sp^2^ C=N (5.2 at %) in EM300 (Figure 2e), which can support above supposition.

In Figure 2a, the N1s XP spectrum of EM300 can be deconvoluted into four configurations, which are assigned to the pyridinic-N (399.0 ± 0.3 eV) [17], nitrile-N (399.4 eV) [13], pyrrolic-N (400.2 ± 0.2 eV) [17,23], and graphitic-N (401.3 ± 0.3 eV) [10], respectively, in the carbon lattice. Their relative percentages are 13.3, 25.5, 40.0 and 21.2 at %, respectively. However, the nitrile-N species is not contained in the three nitrogen-doped carbon materials and is further converted to the pyridinic- and graphitic-N species during high-temperature pyrolysis [27]. Furthermore, the high temperature process also causes the relative proportion of bonding configurations in other carbon materials to be changed. The pyrrolic-N species is rapidly decomposed with the increase of the pyrolysis temperature and its relative percentage largely decreases from 40.6 at % for N-BC@CNP-700 to 28.0 at % for N-BC@CNP-900, which is inconsistent with the results reported by Perazzolo’s group [28]. On the contrary, the proportion of pyridinic- and graphitic-N species is effectively increased 4.1 and 8.5 at %, respectively. However, the relative proportion of pyrrolic-N is increased but the relative proportions of pyridinic-N and graphitic-N are decreased at N-BC@CNP-1000.

### 3.2. Electrocatalytic Activity and Stability

**Figure 3:**
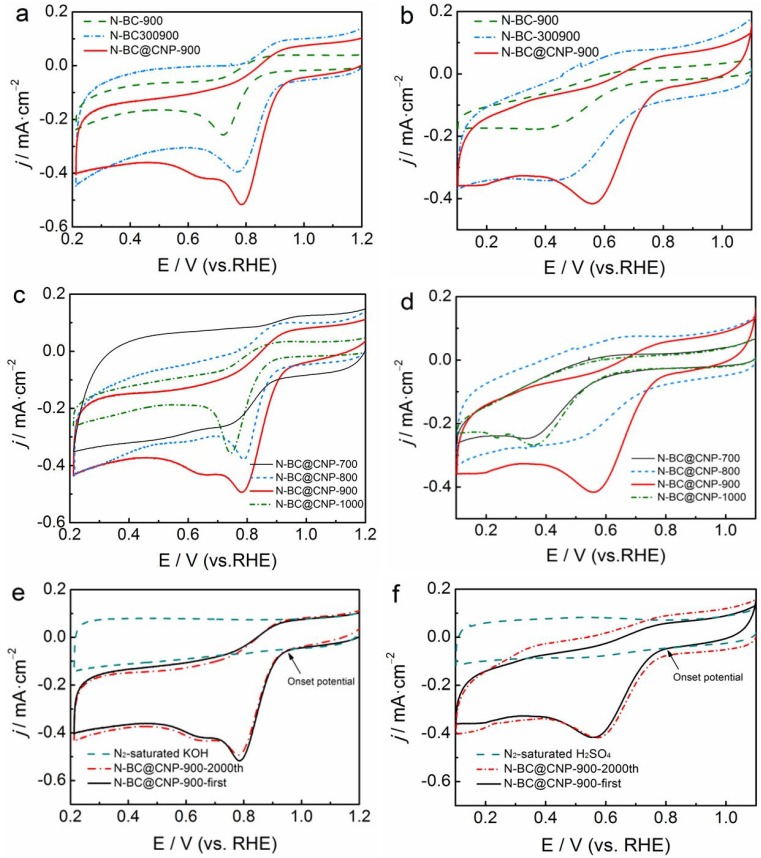
Cyclic voltammograms (CVs) of N-BC-900, N-BC-300900 and N-BC@CNP-900 in O_2_-saturated 0.1 mol·L^−1^ KOH solution (**a**) or 0.5 mol·L^−1^ H_2_SO_4_ solution (**b**); CVs of N-BC@CNP-700, N-BC@CNP-800, N-BC@CNP-900 and N-BC@CNP-1000 in O_2_-saturated 0.1 mol·L^−1^ KOH solution (**c**) or 0.5 mol·L^−1^ H_2_SO_4_ solution (**d**); CVs of N-BC@CNP-900 before and after accelerated aging test (AAT) for oxygen reduction reaction (ORR) in 0.1 mol·L^−1^ KOH (**e**) or 0.5 mol·L^−1^ H_2_SO_4_ (**f**) solutions under O_2_ protection at a scan rate of 5 mV·s^−1^. RHE: reversible hydrogen electrode.

The ORR electrocatalytic activities of N-BC-900, N-BC-300900 and N-BC@CNP-900 were evaluated by CV in 0.1 mol·L^−1^ KOH or 0.5 mol·L^−1^ H_2_SO_4_ solution saturated by oxygen, as indicated in Figure 3a,b. It is found that these materials exhibit the ORR catalytic activity in both alkaline and acidic solutions, but the activity of N-BC-900 from the direct carbonization of EM biomass is relatively poor compared with other carbon-based catalysts. The peak potential for the ORR of N-BC-900 is only 0.72 and 0.42 V in 0.1 mol·L^−1^ KOH and 0.5 mol·L^−1^ H_2_SO_4_ solutions, respectively. N-BC-300900 has displayed better ORR activity with more positive peaks and onset potentials for ORR compared to the N-BC-900. The enhanced peak current density of N-BC-300900 is nearly twice as large as that of N-BC-900. The results demonstrate that the ORR activity of carbon-based catalysts can be effectively improved by transformation of the pyrolysis process from one-step to two-step, which may be attributed to the change of physical characteristics of the carbon matrix, helping the doping of nitrogen into the graphite structure [29]. It is noted that the N-BC@CNP-900 has the highest ORR catalytic activity in aqueous solutions. In alkaline medium, the onset potential and peak potential for the ORR of N-BC@CNP-900 are about 0.94 and 0.79 V, respectively, which are positively shifted about 45 and 16 mV compared to those of N-BC-300900. However, the CV curve of N-BC@CNP-900 has also displayed another weak ORR peak at ~0.65 V in alkaline medium. This phenomenon is more likely due to the irreversible reduction of oxygen on different catalytic sites located on the edges and on the basal plane [30]. In acidic medium, the onset potential and peak potential for ORR of N-BC@CNP-900 are about 0.80 and 0.56 V, respectively, which are positively shifted about 58 and 86 mV compared to those of N-BC-300900. Meanwhile, the ORR peak current density of N-BC@CNP-900 has been obviously enhanced. The differences between N-BC-300900 and N-BC@CNP-900 in the ORR activity are attributed to three aspects: (i) introducing carbon nanoparticle support may produce more exposed catalytically active sites and provide a larger space for more active catalytic sites by preventing agglomeration during pyrolysis; (ii) the addition of carbon nanoparticles helps to form new ORR catalytic sites on the modified carbons with other N-containing fragments derived from further decomposition of EM300 during high-temperature pyrolysis [23]; (iii) the addition of carbon nanoparticles can increase the connectivity between the doped carbon particles and promote the conductivity of the N-BC@CNP-900 electrocatalyst, which may be beneficial to the improvement of its ORR electrocatalytic activity [31].

The influence of pyrolysis temperature (700–1000 °C) on the ORR activity in both alkaline and acidic solutions was further examined in Figure 3c,d. It can be observed that the ORR activity of the carbon material is largely enhanced with the increase of pyrolysis temperature below 900 °C, but it is rapidly decreased while the pyrolysis temperature is 1000 °C. This phenomenon can be ascribed to a reasonable fact that more active sites for the ORR in the catalysts are produced at 900 °C, and a higher or lower pyrolysis temperature can act against the formation of catalytic sites on the surface of the carbon material [24]. Additionally, the ORR peak current densities of four carbon materials are significantly larger in 0.1 mol·L^−1^ KOH solution than in 0.5 mol·L^−1^ H_2_SO_4_ solution. More positive ORR peak potential can be also observed in 0.1 mol·L^−1^ KOH solution. It is significantly related to the improved kinetics of the ORR in alkaline electrolyte compare to in acidic electrolyte. These differences powerfully show that fungus-derived carbon materials possess better ORR activity in alkaline electrolytes.

Long-term durability is another factor to assess whether the prepared carbon-based catalysts can be applied to practical commercialization. We here examine the electrochemical durability of N-BC@CNP-900 by CV continuous scanning for 2000 cycles in N_2_-saturated 0.1 mol·L^−1^ KOH or 0.5 mol·L^−1^ H_2_SO_4_ solutions, as shown in Figure 3e,f. It is observed that all the CVs of N-BC@CNP-900 in an aqueous solution of 0.1 mol·L^−1^ KOH or 0.5 mol·L^−1^ H_2_SO_4_ under N_2_ protection have suggested virtually featureless curves (dash line in Figure 3e,f), in which the ORR peaks cannot be observed. More importantly, the ORR activity of N-BC@CNP-900 has not been decreased before and after the AAT in both alkaline and acidic electrolytes. The peak potential, onset potential, and peak current density for ORR are not obviously changed. The results show that N-BC@CNP-900 has good durability and can be suitable for the catalysis of the ORR under alkaline and acidic conditions.

**Figure 4:**
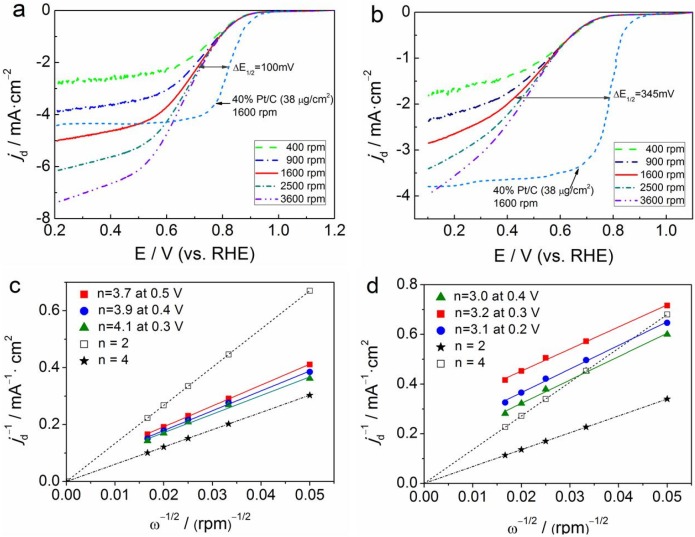
(**a**) The ORR polarization curves of N-BC@CNP-900 in O_2_-saturated 0.1 mol·L^−1^ KOH solution at different rotation rates (400–3600 rpm) and JM Pt/C catalyst in O_2_-saturated 0.1 mol·L^−1^ KOH at 1600 rpm; (**b**) The ORR polarization curves of N-BC@CNP-900 in O_2_-saturated 0.5 mol·L^−1^ H_2_SO_4_ solution at different rotation rates (400−3600 rpm) and JM Pt/C catalyst in O_2_-saturated 0.5 mol·L^−1^ H_2_SO_4_ solution at 1600 rpm; (**c**) Koutecky-Levich plots of *j*_d_^−1^ *vs.* ω^−1/2^ obtained from Figure 4a; (**d**) Koutecky-Levich plots of *j*_d_^−1^ *vs.* ω^−1/2^ obtained from Figure 4b.

In order to study the electrocatalytic mechanism of the ORR on our materials in 0.1 mol·L^−1^ KOH or 0.5 mol·L^−1^ H_2_SO_4_ electrolytes, we used the RDE measurements with different rotation rates (400–3600 rpm) to thoroughly examine the activity of as-prepared N-BC@CNP-900 and commercial Pt/C catalyst (Johnson Matthey) and reveal the kinetics of the ORR in different electrolytes, as shown in Figure 4a,b, respectively. We expectedly find that the disk current density (*j*_d_) of ORR on RDE is directly proportional to the rotation rate but is independent of the pH value of the electrolyte, which should be attributed to a feasible explanation that the increase in the diffusion of oxygen on RDE results in the enhancement of the corresponding diffusion current density of the ORR. Notably, the ORR current density of N-BC@CNP-900 at +0.5 V *vs.* RHE is almost equal to that of the Johnson Matthey (JM) Pt/C catalyst in alkaline medium. The ORR onset potential (*E*_ORR_) of N-BC@CNP-900 also approaches that of the JM Pt/C catalyst (+0.95 V), although the ORR half-wave potential (*E*_1/2_) measured on our material is around 0.73 V in alkaline medium, lower than that on the commercial JM Pt/C catalyst (0.83 V). However, the ORR current density of N-BC@CNP-900 at +0.5 V *vs.* RHE is only 42.8% of the JM Pt/C catalyst and the ORR half-wave potential (*E*_1/2_) of N-BC@CNP-900 is lower than that of the JM Pt/C catalyst at about 345 mV. These results demonstrate that the N-BC@CNP-900 can be more effective to catalyze the ORR in alkaline medium to a certain degree. In contrast to the ORR curves at JM Pt/C, the ORR curves at N-BC@CNP-900 do not level off as expected at large over-potentials of O_2_ reduction. There is no well-defined diffusion limiting current plateau at any rotation rate. It was considered to be an artifact of the porous coating electrode due to roughness or hydrodynamic flow in the porous coating caused by the pressure gradient across the face of the disk [32].

The Koutecky-Levich (K-L) theory was applied to further calculate the overall electron transfer numbers (*n*) and kinetic current density (*j*_k_) of the ORR based on the fact that the current densities are dependent on the electrode rotation rates in Figure 4a,b. The diffusion-limited current density (*j*_d_) on an RDE was estimated by the K-L equation (Equation (1)) [33].

(1) $$\frac{1}{j_{\text{d}}}=\frac{1}{j_{\text{k}}}+\frac{1}{0.62nFC_{\text{O}}D_{\text{O}}^{2/3}v^{-1/6}\omega^{1/2}}$$

where *F* is the Faradaic constant (C·mol^−1^); *C*_O_ is the O_2_ saturation concentration in the aqueous solution (mol·cm^−3^); *D*_O_ is the O_2_ diffusion coefficient in the aqueous solution (cm^2^·s^−1^); *v* is the kinetic viscosity of the solution (cm^2^·s^−1^); and ω is the electrode rotation rate (rpm). The K-L plots of *j*_d_^−1^ *versus* ω^−1/2^ for N-BC@CNP-900 in 0.1 mol·L^−1^ KOH and 0.5 mol·L^−1^ H_2_SO_4_ electrolytes are shown in Figure 4c,d, respectively. As a control, the theoretical K-L curves of two electrons and four electrons in full-diffusion controlled conditions were also plotted in Figure 4c,d. The linearity and parallelism of all K-L plots also indicate consistent electron transfer at different potentials and first-order reaction kinetics with respect to the concentration of dissolved oxygen. The average number of electron transfer is calculated to be ~3.9 at 0.3–0.5 V in alkaline medium and ~3.1 at 0.2–0.4 V in acidic medium from the slopes (1/0.62*nFC*_O_*D*_O_^2/3^*ν*^−1/6^) of K-L plots, respectively. It suggests that the ORR catalyzed by N-BC@CNP-900 can involve a mixture of two- and four-electron transfer pathways but is a predominant four-electron transfer pathway to produce H_2_O [23]. Besides, the average kinetic current densities obtained from the intercepts (1/*j*_k_) of K-L plots are 21.7 mA·cm^−2^ at 0.3–0.5 V and 3.6 mA·cm^−2^ at 0.2–0.4 V for alkaline and acidic conditions, separately. For this reason, we further confirm that N-BC@CNP-900 should be more effective for the ORR electrocatalysis in alkaline medium because of a higher kinetic current density and larger electron transfer number and is expected to function as one of the alternatives to commercial Pt-based catalysts.

On the basis of XPS analysis and electrochemical results, it can be concluded that a higher percentage of the pyrrolic-N species does not enhance the ORR electrocatalytic activity, whereas a higher percentage of pyridinic- and graphitic-N species can effectively improve the ORR catalytic performance in both alkaline and acidic electrolytes, which can be the catalytically active site structures of our carbon materials for ORR [30,34]. More significantly, the pyridinic-N species can be well retained under the condition of high temperature and its percentage is always higher than that of the graphitic-N species in the prepared materials. We can reasonably conclude that the pyridinic-nitrogen can play a key role in stabilizing the ORR activity, but the graphitic-nitrogen can enhance the ORR activity only. This view on the active centers is in good agreement with the reported results from the literature [10,35,36].

## 4. Conclusions

In this work, a new nitrogen-containing carbon-based electrocatalyst for oxygen reduction was originally designed by an all-solid-state pyrolysis method with the usage of bioprotein-rich enoki mushroom biomass and cheap carbon nanoparticles as the starting materials. It exhibits the electrocatalytic activity, high durability, and selectivity for the four-electron oxygen reduction pathway. The onset potential of ORR on as-prepared N-BC@CNP-900 is around 0.94 V and the number of the electron transfer on N-BC@CNP-900 is ~3.9, approaching those of commercial Pt/C catalyst in alkaline medium. Besides, we importantly proposed that the pyridinic- and graphitic-nitrogen functional groups can play a pivotal role in improving the ORR activity of carbon materials, and may be the electrocatalytic site structures for ORR in both alkaline and acidic electrolytes. More remarkably, the pyridinic-nitrogen group may stabilize the ORR electrocatalytic activity, but the graphitic-nitrogen group may play a key role in the enhancement of the ORR activity of carbon material. Our results will help researchers in the search for cheap and high-efficiency doped-carbon catalysts for ORR in fuel cells by using abundant and edible fungus biomass from nature as the nitrogen/carbon precursor.

## Supplementary Materials

The following are available online at www.mdpi.com/1996-1944/9/1/1/s1, Figure S1: The TG-DTG analysis of EM dried powder; Figure S2: The SEM image (a,b) of the N-BC@CNP-900 catalyst.
