# Supplementary material for: The Use of an Edible Mushroom-Derived Renewable Carbon Material as a Highly Stable Electrocatalyst towards Four-Electron Oxygen Reduction
Source: Materials (Basel). 2015 Dec 23;9(1):1. doi: 10.3390/ma9010001 (PMC5456573; doi:10.3390/ma9010001)
Supplement: Supplementary file 1 [file materials-09-00001-s001.pdf]

Supplementary Materials: The Use of an Edible Mushroom-Derived Renewable Carbon Material as a Highly Stable Electrocatalyst towards Four-Electron Oxygen Reduction

Chaozhong Guo, Lingtao Sun, Wenli Liao and Zhongbin Li

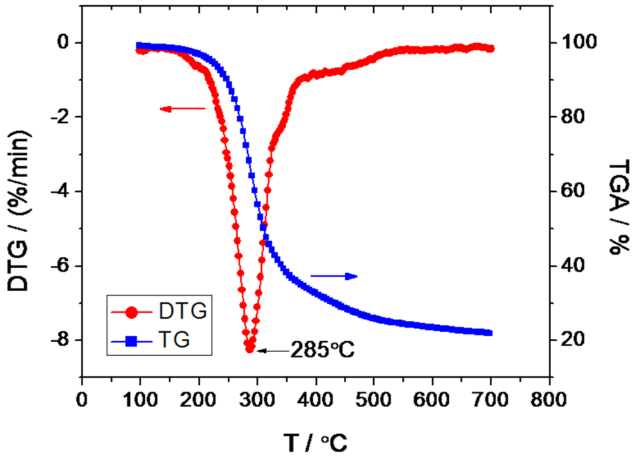

Figure S1. The Thermogravimetric and differential thermogravimetric (TG-DTG) analysis of enoki mushroom (EM) dried powder; TG measurements were recorded from 100 to 700 °C with a ramp rate of 10 °C·min^{-1}. It is found that the decomposition of biological protein inside EM occurred at about 300 °C.

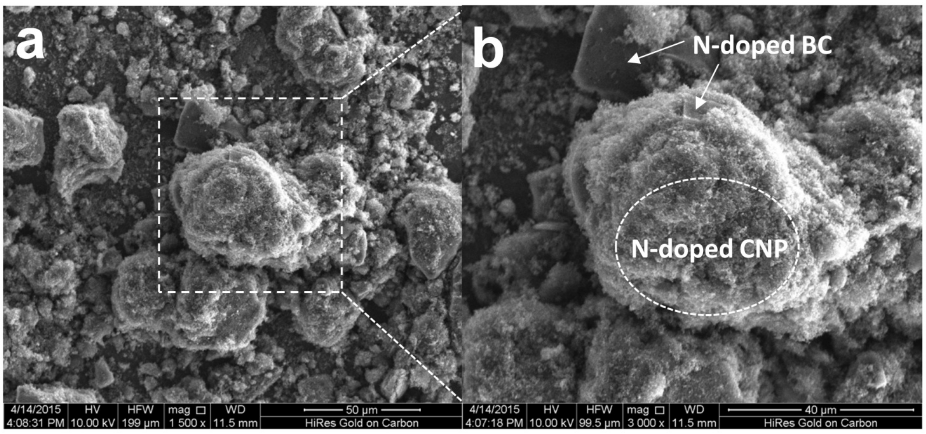

Figure S2. The scanning electron microscopy (SEM) image (**a**,**b**) of the nitrogen-doped biocarbon composite material (N-BC@CNP-900) catalyst.
